# Respiratory Motion‐Corrected Model‐Based 3D Water‐Fat MRA of the Thorax at 0.55 T

**DOI:** 10.1002/mrm.70285

**Published:** 2026-02-04

**Authors:** Robert Stoll, Christoph Kolbitsch, Michaela Schmidt, Marcel Dominik Nickel, Tobias Schaeffter, Daniel Giese

**Affiliations:** ^1^ Department of Medical Engineering Technical University Berlin Berlin Germany; ^2^ Research & Clinical Translation, Magnetic Resonance, Siemens Healthineers AG Erlangen Germany; ^3^ Physikalisch‐Technische Bundesanstalt (PTB) Braunschweig and Berlin Germany; ^4^ Einstein Center Digital Future (ECDF) Berlin Germany

**Keywords:** 0.55 T, cardiac MRA, motion‐correction, water‐fat separation

## Abstract

**Purpose:**

The goal of this study was to develop a 5‐min 3D MRA acquisition at 0.55 T with predictable scan time, 100% data efficiency, and robust water‐fat separation.

**Methods:**

For full data efficiency, the proposed method combined self‐gating with retrospective motion correction while ensuring a predictable 5‐min scan time. Water‐fat separation was implemented using a model‐based Dixon reconstruction. Evaluation in 18 volunteers compared results to navigator‐gated reference scans with nominal scan times of 5 and 10 min via a Likert scale blinded expert rating. Susceptibility to irregular breathing patterns was also analyzed.

**Results:**

The expert rating for image quality was 4.22 for the proposed method, 3.89 for the 5‐min navigator‐gated scan and 4.43 for the 10‐min navigator‐gated scan. Ranking the three methods revealed moderate inter‐rater reliability of 0.46, suggesting only minor differences. While navigator‐gated acquisitions deviated from the expected scan time by −2.26 to 2.86 min and −3.91 to 4.54 min for the 5‐ and 10‐min protocols respectively, the proposed method deviated only by −0.17 to 0.45 min. The self‐gated method further avoided saturation artifacts from the cross‐beam navigator, allowing better distinction of the right pulmonary veins. Image quality for the proposed method was also less susceptible to irregular breathing patterns.

**Conclusion:**

Whole‐thorax MRA acquisitions with water‐fat separation and predictable scan times were successfully acquired in 18 volunteers at 0.55 T. The proposed method demonstrated on average better image quality than navigator‐gated acquisitions of the same nominal scan time while mitigating limitations of prospective navigator gating.

## 1 Introduction

Given its importance in interventional follow‐ups and preventive vascular assessments, magnetic resonance angiography (MRA) is a key imaging tool, particularly for conditions such as aortic disease and congenital heart disease. In patients with renal disease, non‐contrast‐enhanced (non‐CE) MRA is especially valuable, as it eliminates the risks associated with gadolinium‐based contrast agents [1].

MRA at lower field strengths, such as 0.55 T, is of increasing interest, improving accessibility and applicability. With the intrinsic lower signal‐to‐noise ratios at lower field strengths, however, acquisition times are often increased. Additionally given the demand for clinically feasible resolutions and large coverages, high acceleration factors are required. Recently, this has been achieved for MRA [2, 3], by using large undersampling factors in combination with a Compressed Sensing reconstruction.

As MRA must typically be acquired during free‐breathing, robust breathing motion compensation remains a major challenge. Respiratory motion can be tracked using MR‐navigators positioned at the liver/diaphragm interface in combination with a prospective accept/reject algorithm [4]. These techniques can however lead to signal saturation artifacts, potentially obscuring vascular structures such as the right pulmonary veins [5, 6]. Furthermore, the scan duration is unpredictable as it depends on the patient's respiration pattern. Approaches to tackle these shortcomings have been proposed, such as self‐gating methods or image‐based navigators in combination with retrospective motion correction [7].

Additionally, fat separation or saturation is typically required for MRA acquisitions. When transitioning to lower field strengths, however, the spectral proximity of water and fat makes spectral fat saturation techniques challenging. A recently proposed method [8] used Bloch simulations to optimize fat saturation for MRA at 0.55 T in combination with a balanced SSFP (bSSFP) readout and image‐based navigators, achieving a 3‐fold undersampling. The authors discussed that residual fat signal was visible, and a smaller coil was used to restrict the field‐of‐view. As an alternative, Dixon‐based water‐fat separation [9] has been applied to MRA at 0.55 T, additionally incorporating T1 and T2 mapping [10] also using an image‐based navigator for motion compensation, achieving a 4‐fold undersampling. In classical Dixon algorithms, echo images are usually reconstructed before being unfolded into fat and water images. On the other hand, model‐based Dixon algorithms [11, 12] allow regularization to be performed on the fat and water images directly and ensure data consistency of the water‐fat images with the acquired data. Combining Compressed Sensing with model‐based Dixon has been applied for 3D abdominal imaging [13] as well as in cardiac and respiratory 3D cardiac MRI [14].

In the present work, we propose a whole‐thorax Dixon‐based MRA sequence at 0.55 T with a predictable scan time of 5 min (SELF5). An empirically optimized sampling scheme was developed to allow for retrospective respiratory motion correction [15] based on a $k_{y}=k_{z}=0$ self‐navigator. A dual‐echo real‐valued water‐fat model was integrated into a Compressed Sensing‐based Reconstruction [16, 17], allowing undersampling factors of ∼7.8.

The proposed method SELF5 was evaluated in 18 volunteers and compared to reference navigator‐gated sequences with expected 5‐min (NAV5) and 10‐min (NAV10) acquisitions [2], by analyzing image quality and its breathing pattern dependence as well as scan times.

## 2 Methods

### 2.1 Data Acquisition

**FIGURE 1:**
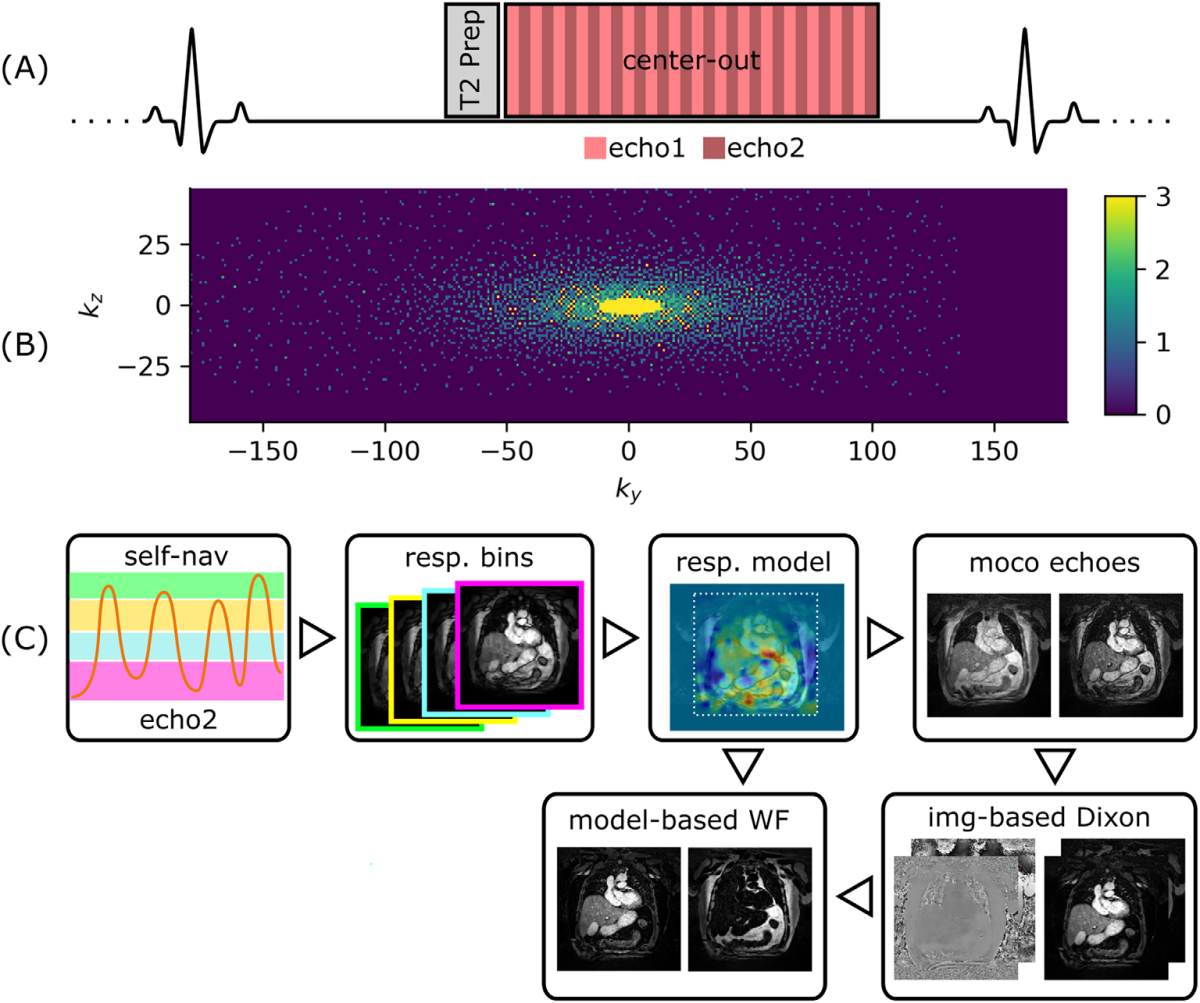
Schematic of the acquisition and reconstruction process for SELF5. (A) Data is acquired during late diastole in a center‐out fashion after T2 preparation. (B) K‐space data is distributed, such that a focus is put on the central region, while a minimum sampling density is enforced in the periphery. (C) For the reconstruction, a surrogate respiratory signal is derived from the central k‐space lines. This is used for binning and a respiratory‐resolved reconstruction of the second echo, allowing for the derivation of motion fields T. A preliminary motion corrected reconstruction of the echoes is fed into an image‐based Dixon algorithm, yielding water‐fat images and a set of error phasors B. T, water‐fat images and B are passed to the final model‐based reconstruction, which directly yields fat and water images.

All research sequences were applied on 18 volunteers after informed consent was given at 0.55 T (MAGNETOM Free.Max, Siemens Healthineers, Forchheim, Germany) using a 12‐channel anterior and 6‐channel posterior array coil. Cardiac gating was achieved with an external triggering device (Expression MR400, Philips NV). For increased blood/myocardium contrast, a 40 ms T2‐preparation pulse was used. Two echoes per k‐space line were acquired (TE = 2.8/6.5 ms) using a bipolar readout gradient (Figure 1A). The resulting TR was 9.1 ms, a flip angle of 15° and a bandwidth of 401 Hz/pixel were chosen. A partial Fourier factor of 0.875 was applied in both phase encoding dimensions. The field‐of‐view (FoV) was set to cover the entire thoracic vasculature, with 450 × 450 × 125 mm^3^, and an acquired isotropic resolution of 1.5 mm. The number of k‐space lines per ECG cycle was set to 16 or 13, depending on heart rates, corresponding to acquisition windows of 145 or 118 ms.

Data was acquired using an ECG‐triggered dual‐echo spoiled gradient echo sequence during the quiescent phase in diastole (Figure 1A) based on a 4‐chamber cine acquisition. A k‐space sampling scheme on a Cartesian grid was employed, with the readout dimension along the feet‐head direction. The distribution in the phase encoding dimensions consisted of an overlay of three variable density Poisson Disk sampling patterns with differing random seeds and cropped corners. Each pattern enforced full k‐space coverage for the central 5% of the normalized radius and a minimum sampling density at the periphery (Figure 1B). For the final sampling point distribution of SELF5, the number of individual sampling patterns, the fully sampled central region and the density distribution were empirically optimized. Adding a fully sampled region of 5% was deemed sufficient to trade‐off central k‐space coverage of each motion state after binning with the density of k‐space periphery. Restricting the number of patterns to 2 resulted in insufficient data per motion bin, while 4 patterns did not substantially improve the motion field estimation while losing details in the final reconstruction compared to 3 patterns. Adding a minimum sampling density further ensured peripheral coverage.

In preparation for the reordering, the sampling points were divided with respect to their normalized radius into $n_{\text{segments}}-1$ bins, where $n_{\text{segments}}$ corresponds to the number of k‐space lines acquired per ECG cycle. During each acquisition window, the central k‐space line followed by lines from each bin were acquired in a center‐out fashion.

The proposed method was setup to achieve a 5‐min acquisition time, assuming a 60‐bpm heart rate (SELF5). Similarly, the navigator‐gated reference method was setup to achieve a 5‐min acquisition time, assuming a respiratory acceptance rate of 50% (NAV5). An additional navigator‐gated acquisition was setup to achieve a 10‐min acquisition time, assuming a respiratory acceptance rate of 50% (NAV10). This acquisition was used as a ground truth reference, as its number of acquired k‐space points match the number of k‐space points of SELF5, while doubling scan time. The total acceleration rates were ∼7.8 for SELF5, ∼11.5 for NAV5 and ∼5.7 for NAV10. The prospective navigator gating for NAV5 and NAV10 was configured as a cross‐beam diaphragm navigator with an acceptance window of ±4 mm.

### 2.2 Motion Compensation

The entire reconstruction pipeline [18] is schematically depicted in Figure 1C. First, a respiratory self‐navigation signal was extracted from the central k‐space lines ($k_{y}=k_{z}=0$) of each acquisition window by applying a principal component analysis along the readout and coil dimensions. The largest singular value was associated with the respiratory motion signal. The surrogate signal was then spline‐interpolated and used to bin the k‐space data into m=4 respiratory motion states. The split was performed based on the magnitude of the surrogate signal, ensuring a similar number of phase‐encoding points in each bin and no data sharing was employed.

A respiratory‐resolved reconstruction was then performed on the second (opposed phase) echo with the optimization problem being

$$\min_{s_{2}}\;\sum_{m}{\left\|E_{m}(s_{m,2}\}-k_{m,2}\right\|}_{2}^{2}+\lambda_{\mathrm{TV}}{\left\|\nabla_{x}(s_{2})\right\|}_{1}+\lambda_{\mathrm{TVT}}{\left\|\nabla_{t}\left(s_{2}\right)\right\|}_{1},$$

(1) $$E_{m}\left(s_{m,2}\right)=F\left(C\circ s_{m,2}\right).$$

Here, $s_{2}$ indicates the image of the acquired second echo (split into m motion states), $k_{2}$ indicates the acquired k‐space data of the second echo (split into m motion states), $\nabla_{x}$ the finite difference operator along the spatial dimensions, $\nabla_{t}$ the finite difference operator along the respiratory dimension, $\lambda_{\mathrm{TV}}$ and $\lambda_{\mathrm{TVT}}$ their respective regularization weights, E the Cartesian encoding operator, F the Fourier operator and C the coil sensitivities. The respiratory‐resolved reconstruction was performed over 15 iterations using the limited‐memory Broyden‐Fletcher‐Goldfarb‐Shanno (LBFGS) algorithm [19], with regularization parameters $\lambda_{\mathrm{TV}}=\lambda_{\mathrm{TVT}}=0.001$.

3D respiratory motion fields T were derived from these respiratory‐resolved images via a non‐rigid registration. Motion fields were computed using the Medical Image Registration ToolKit (MIRTK) [20]. A spline support point distance of six voxels was used, constraining the transformation over larger regions and thereby enforcing a smoother deformation while avoiding overfitting on noise. To enhance computational efficiency, a rectangular region of interest (ROI) was manually defined to constrain motion fields to the torso. A Gaussian decay was applied at the ROI boundaries to prevent related sharp edges in the final reconstruction.

Although four respiratory bins were sufficient to avoid intra‐bin motion in the heart in seven of the volunteers, some breathing patterns required additional motion bins. Therefore, the motion fields were used to estimate intra‐bin motion in a 4.5 × 4.5 × 4.5 mm^3^ ROI at the liver dome and extrapolate cardiac motion via a scaling factor of 0.57 [21]. Whenever the difference between adjacent motion states ${∆}_{\mathrm{ms}}$ exceeded sub‐voxel displacements, $\left\lfloor {∆}_{\mathrm{ms}}/2\right\rfloor$ intermediate motion states were introduced. The new motion fields were generated through linear interpolation, and the motion bins were updated to ensure minimal distance to the mean surrogate value of the motion state.

### 2.3 Reconstruction of Water‐Fat Separated Images

Since model‐based water‐fat reconstruction poses an ill‐conditioned optimization problem, a good starting value is needed. Therefore, a motion‐compensated conjugate gradient SENSE [22] reconstruction of both echoes was performed, solving the optimization problem

$$\min_{s_{\text{moco}}}\;\sum_{i}\;\sum_{m}{\left\|E_{\text{moco},m,i}\left(s_{\text{moco},i}\right)-k_{m,i}\right\|}_{2}^{2},$$

(2) $$E_{\text{moco},m,i}\left(s_{\text{moco},i}\right)=P_{m}\circ F\left(T_{m}\circ C\circ s_{\text{moco},i}\right).$$

Here, i is the echo index, $s_{\text{moco}}$ the motion‐corrected echo images, $E_{\text{moco}}$ the Cartesian encoding operator with motion correction, m the motion state index, $P_{m}$ a masking operator limiting the k‐space data to their respective motion state, and $T_{m}$ the transformation to warp the image to the motion state m.

Using $s_{\text{moco}}$ in conjunction with a spectral fat model $d_{\mathrm{fat}}=\left\{d_{\mathrm{fat},1},d_{\mathrm{fat},2}\right\}$, fat and water separated images as well as error phasors B were obtained via an image‐based water‐fat separation algorithm based on the graph‐cut algorithm [2, 23]. B consists of $b_{1}$ being the phase of the water signal for the first echo, while $b_{2}$ additionally includes phase effects between the echoes due to effects such as $B_{0}$ inhomogeneities and eddy currents. The signal model was defined as

$$s_{\text{moco},1}=\left(w+d_{\mathrm{fat},1}\cdot f\right)\circ b_{1},$$

(3) $$s_{\text{moco},2}=\left(w+d_{\mathrm{fat},2}\cdot f\right)\circ b_{2},$$

with w and f being the real‐valued water and fat image vectors, $d_{\mathrm{fat},i}=\sum_{s}\alpha_{s}\exp\left(2\mathrm{\pi j\Delta}f_{s}TE_{i}\right)$ are complex scalars describing the temporal evolution of the fat signal, with $\Delta f_{s}$ being the frequency shift between water and fat for spectral peak s, α their respective weights and TE the echo times. $d_{\mathrm{fat}}$ was empirically calibrated for the echo times of ∼2.8 and ∼6.5 ms as ∼0.70–0.37j and ∼−0.58–0.20j respectively. B was smoothed using Gaussian kernels along each spatial dimension with a standard deviation of 1.0 and a radius of 4, while preserving unit‐vector properties and the original difference between $b_{1}$ and $b_{2}$. Here, water and fat signals were modeled as real‐valued with a common phase. This has been shown to increase the robustness to noise, decrease the number of unknowns in the signal model, avoid the in‐ and opposed phase constraint for 2‐point approaches and thereby enable a faster and more flexible acquisition [24, 25, 26].

For the final motion‐corrected model‐based water‐fat reconstruction, the above signal model was incorporated into the iterative reconstruction process, w and f computed in the previous step were used as a starting value and B was reused. T and B remained unchanged throughout iterations. The corresponding reconstruction problem and encoding operator E are given as

$$\min_{\left(w,f\right)}\;\sum_{i}\;\sum_{m}{\left\|E_{m,i}\left(w,f\right)-k_{m,i}\right\|}_{2}^{2}+\lambda_{\mathrm{TV},w}{\left\|\nabla_{x}\left(w\right)\right\|}_{1}+\lambda_{\mathrm{TV},f}{\left\|\nabla_{x}\left(f\right)\right\|}_{1},$$

(4) $$E_{m,i}\left(w,f\right)=E_{\text{moco},m,i}\left(b_{i}\circ w\right)+d_{\mathrm{fat},i}\;E_{\text{moco},m}\left(b_{i}\circ f\right),$$

where $\lambda_{\mathrm{TV},w}$ and $\lambda_{\mathrm{TV},f}$ denote the strength of the TV regularization of the water image and the fat image respectively.

The final model‐based reconstruction was executed using 10 LBFGS iterations with $\lambda_{\mathrm{TV},w}=\lambda_{\mathrm{TV},f}=0.0007$. For a better comparison to SELF5, NAV5 and NAV10 reconstructions were performed using the same pipeline without motion correction ($\lambda_{\mathrm{TV},w}=\lambda_{\mathrm{TV},f}=0.0007$ and $\lambda_{\mathrm{TV},w}=\lambda_{\mathrm{TV},f}=0.001$ respectively). None of the reconstructions used distortion corrections due to gradient non‐linearities. Regularizations were tuned empirically for each method to be as small as possible, while on average reaching the target maximum iteration. Insufficiently large updates of the objective function meant convergence before reaching the maximum number of iterations.

### 2.4 Data Analysis

In addition to qualitative comparisons, the data was rated by two experts (25 and 15 years of CMR experience). The following ratings were performed on Likert scales: image quality (ignoring water‐fat swaps) (1: non‐diagnostic, 2: poor, 3: moderate, 4: good, 5: excellent), quality of water‐fat separation (1: large swaps, 2: small swaps, 3: no swaps) and myocardial sharpness (1: low, 2: moderate, 3: very sharp). The data was reviewed in 3D using interactive multi‐plane reformatting on both water and fat datasets. Fat images were mainly used to validate water‐fat swaps.

The same data was additionally evaluated in an intra‐volunteer scoring for which the liver/diaphragm was masked on all datasets, to avoid bias. The raters were asked to rank the three images, for relative image quality (ignoring water‐fat swaps) (1: best, 2: mid, 3: worst) and relative quality of the water‐fat separation (1: least number of swaps, 2: mid number of swaps, 3: highest number of swaps). In this comparative ranking, on‐par was allowed (e.g., 1/1/1, 1/1/2, or 1/2/2). For later evaluation, the rankings r were transformed to reflect how much better a method was rated compared to the others within each volunteer v.

$$r_{v,\text{method},\mathrm{new}}=\max_{\text{method}}\left(r_{v,\text{method}}\right)-r_{v,\text{method}}$$

Rankings were combined and rounded to the closest integer. Rater 1 was given a weight of 1.1 and expert 2 a weight of 0.9 to resolve tiebreakers. Inter‐rater reliability was determined using quadratically weighted Cohen's Kappa and statistical significance was evaluated via a Friedman test, followed by a paired Wilcoxon test with Holm‐Bonferroni correction.

To assess the impact of the respiratory pattern on the final reconstruction result, the respiratory motion was compared between SELF5, NAV5, and NAV10. For the navigator‐gated approach, the acceptance (end‐expiratory) position in millimeters was used. In the case of SELF5, the respiratory surrogate signal was scaled by estimating the displacement of the liver/diaphragm interface via the motion fields and accounting for the intra‐bin motion range, yielding the entire respiratory range. The accepted respiratory position was evaluated as a histogram.

## 3 Results

Baring a repeat acquisition of volunteer 7 due to bulk motion, all acquisitions were successful and were included in the analysis. The deviation from the target scan time predicted at the scanner in minutes (mean ± standard deviation; [minimum, maximum]) were 0.03 ± 0.13 [−0.17, 0.45] for SELF5, −0.03 ± 1.22 [−2.26, 2.86] for NAV5 and −0.28 ± 2.14 [−3.91, 4.54] for NAV10, which has double the nominal scan duration. While the mean deviations for NAV5 and NAV10 were small, they exhibited larger standard deviations. The respiratory acceptance rates were 48.8% ± 8.6% [32.2, 67.1] for NAV5 and 51.8% ± 8.5% [37.6, 67.4] for NAV10. By design, SELF5 achieved 100% data efficiency.

**FIGURE 2:**
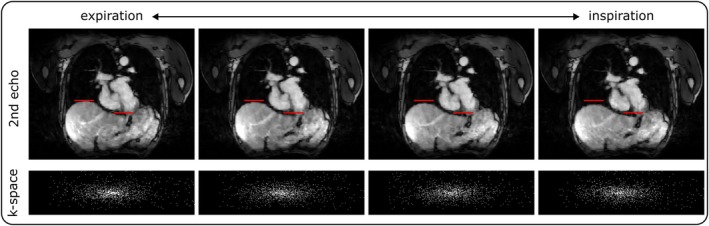
Depiction of the respiratory‐resolved reconstruction in 4 motion bins. During expiration, the heart and liver are shifted upwards compared to the inspiratory state. The images appear blurry due to stronger regularization needed for the higher undersampling. The respective binary k‐space distribution is illustrated below each motion state and shows a focus on the central region.

**FIGURE 3:**
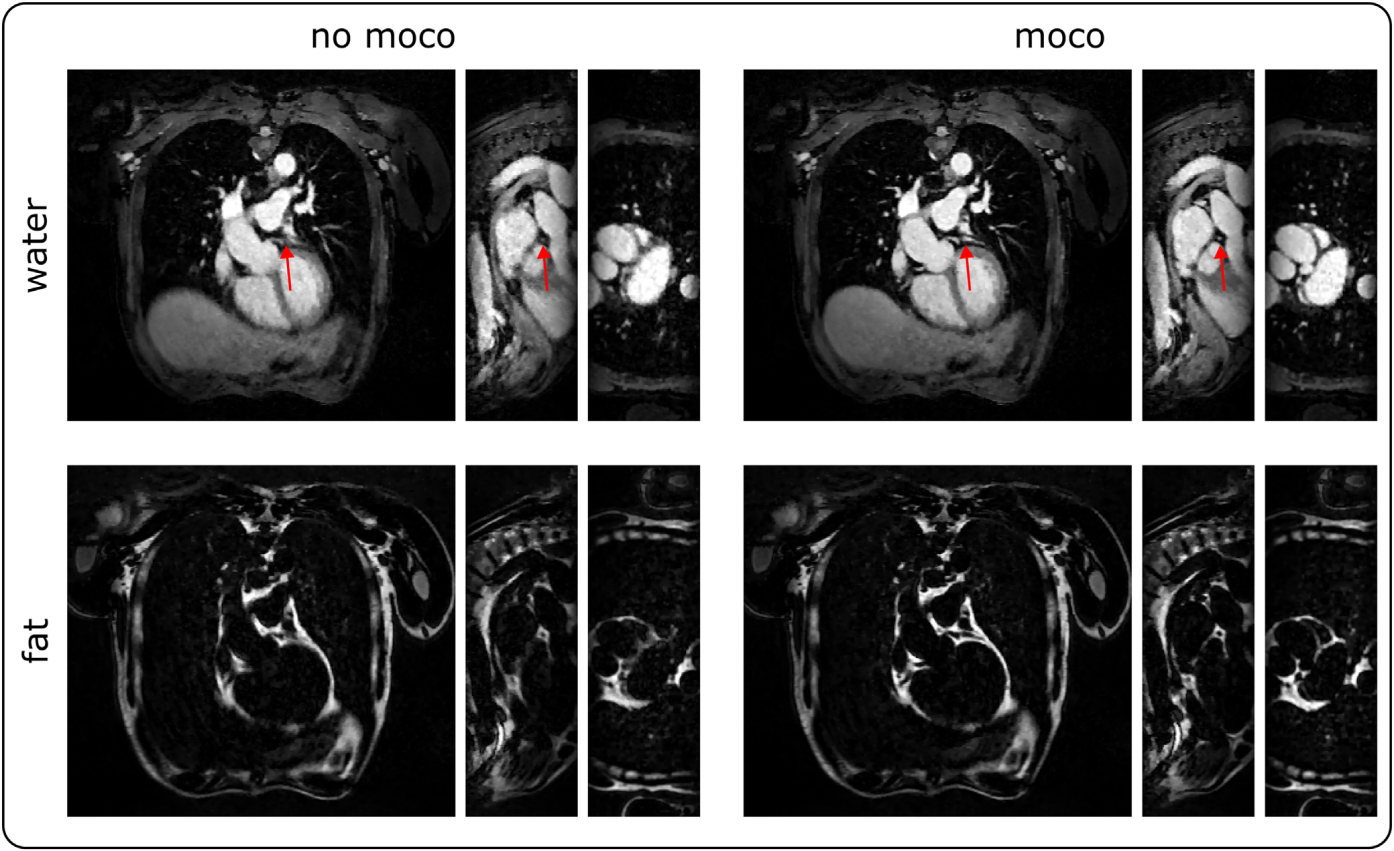
Comparison of SELF5 reconstruction results without (left) and with (right) motion correction. The application of motion correction enhances image sharpness and improves the effectiveness of water‐fat separation. While this effect is observable for all moving structures, it is particularly pronounced in smaller vessels, as demonstrated by the improved visualization of the coronary artery (indicated by red arrows).

The surrogate respiratory signal allowed for binning the data into motion states and calculating the respiratory‐resolved second echo volume, which was used to extract the motion fields T. The four motion states are illustrated in Figure 2 in an exemplary volunteer. In addition, the binary k‐space masks exhibiting high coverage of the central k‐space region across all motion bins are shown. For the volunteer shown, the acceleration in the central 40% of k‐space was around 6.5. The corresponding motion fields for this and two additional volunteers are shown in Video S1. The range of respiratory motion for SELF5 was 20.04 ± 6.8 mm across volunteers. To demonstrate the impact of motion correction (moco), Figure 3 illustrates the output of the reconstruction pipeline with and without motion correction. The respiratory motion range across volunteers for NAV5 was 8 ± 2.85 mm and for NAV10 it was 9.22 ± 4.38 mm. Note that this only considers accepted end‐expiratory motion states.

**FIGURE 4:**
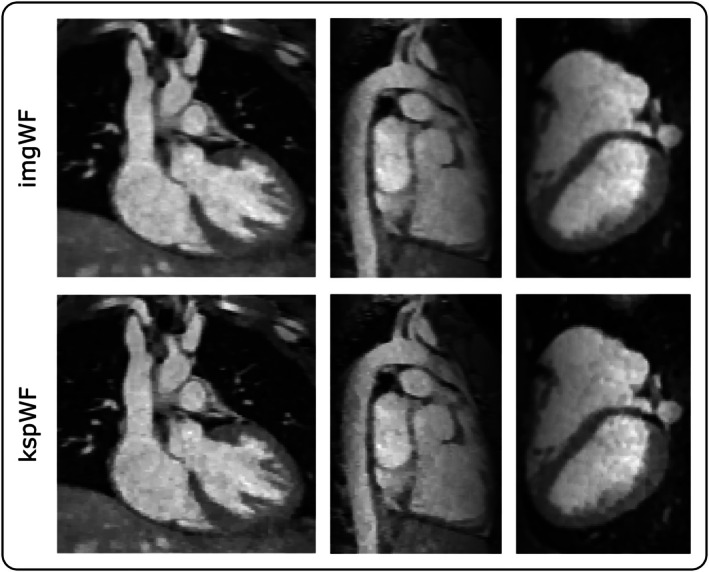
Comparison of SELF5 data using image‐based water‐fat separation (imgWF) and k‐space‐based water‐fat separation (kspWF). It can be observed that the image sharpness and contrast for kspWF are higher than for imgWF.

The model‐based reconstruction started with the result of the image‐based water‐fat separation and improved image sharpness over iterations. In Figure 4 the image‐based reconstruction is compared to the model‐based reconstruction result. Better image sharpness and contrast can be observed for the model‐based approach.

Video S2 shows the final water‐fat reconstructions for all acquired volunteers demonstrating high image quality. The data for volunteer 7 is suspected to contain motion, thus slightly reducing image quality. Volunteer 9 had additional systoles which impair image quality across methods. For volunteer 14, major water‐fat swaps were observed across methods, likely due to blood flow.

**FIGURE 5:**
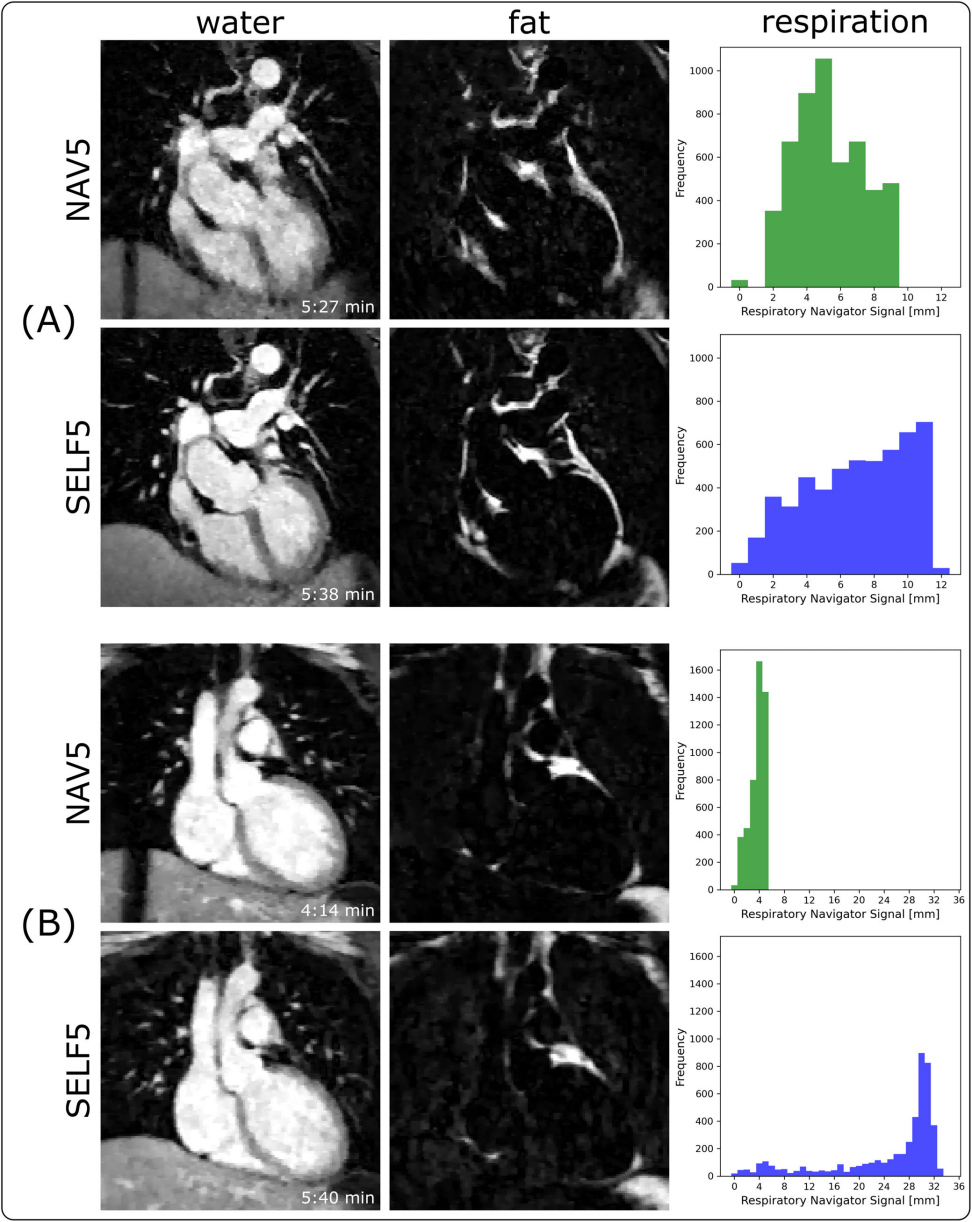
Example comparison of NAV5 and SELF5. (A) shows a case where SELF5 has better ratings for image quality (SELF5 > NAV5). (B) highlights a case, where the SELF5 performed worse for image quality (NAV5 > SELF5). For each case, the respective histogram of the accepted respiratory motion is included to the right.

Figure 5 compares water and fat images between SELF5 and NAV5, highlighting a case where SELF5 ranked better (A) and worse (B). The actual scan durations are displayed in the bottom right of the water images. For each case, the range of accepted respiratory motion is also shown as histograms in Figure 5. Larger motion ranges in the end‐expiratory state, often caused by irregular breathing patterns, were found to degrade the reconstruction quality of NAV5. In contrast, SELF5 exhibited robustness to both wide respiratory motion ranges and variations in end‐expiratory position.

**FIGURE 6:**
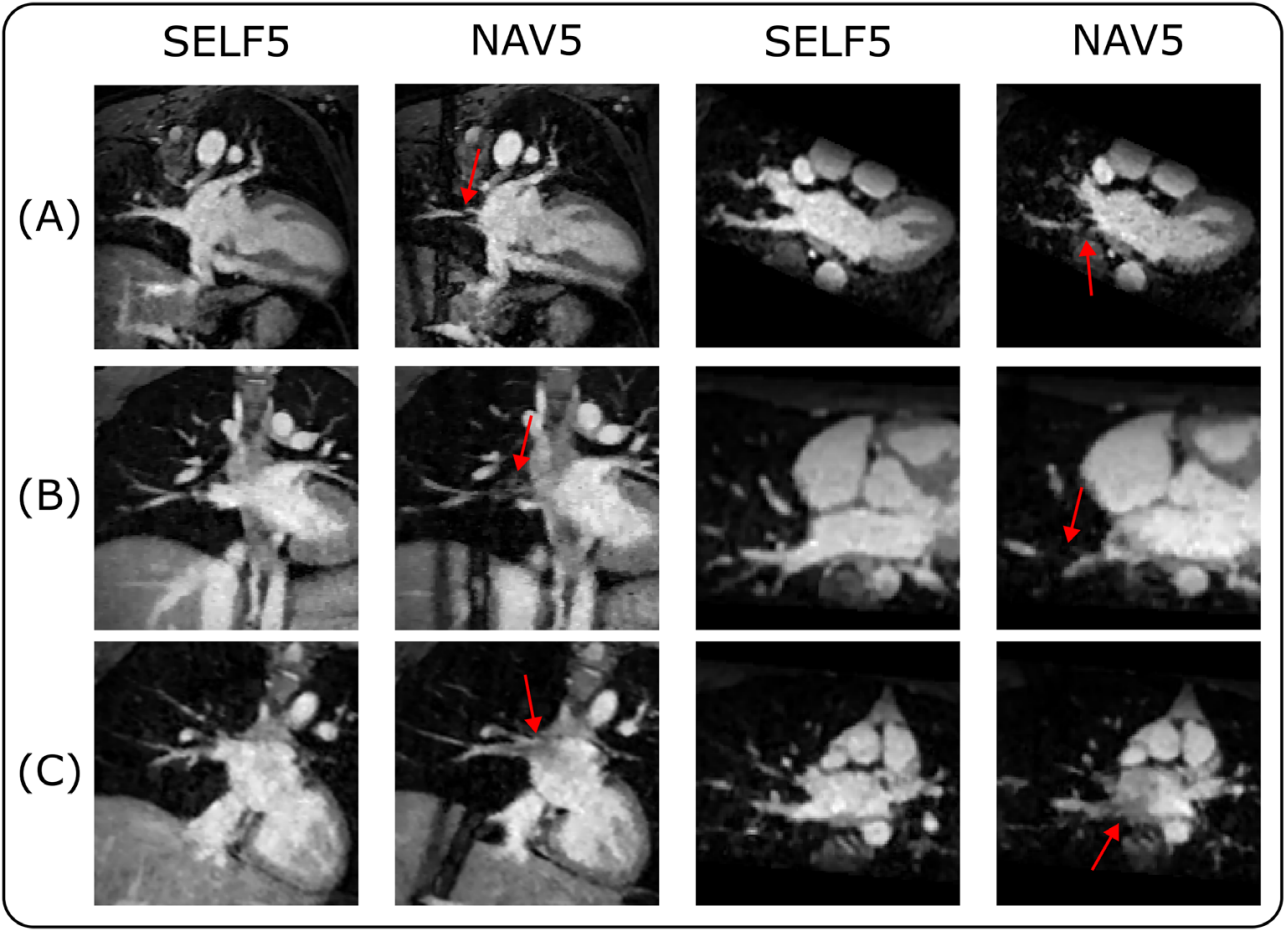
Example comparison of NAV5 and SELF5 in three different volunteers (A, B, C), highlighting the saturation effect in the right pulmonary veins (and atrium) due to the navigator‐pulse for NAV5.

Additionally, the depiction of the right pulmonary veins was assessed across acquisitions. Figure 6 presents a comparison between SELF5 and NAV5 for three representative cases. SELF5 inherently did not exhibit signal saturation artifacts, leading to improved visibility of the right pulmonary veins. In general, vessels crossing the saturation region appeared obstructed, and in some cases, signal voids were observed further along the flow direction due to the upstream ‘tagging’ effect. While Figure 6 specifically illustrates NAV5, similar findings were observed for NAV10.

**FIGURE 7:**
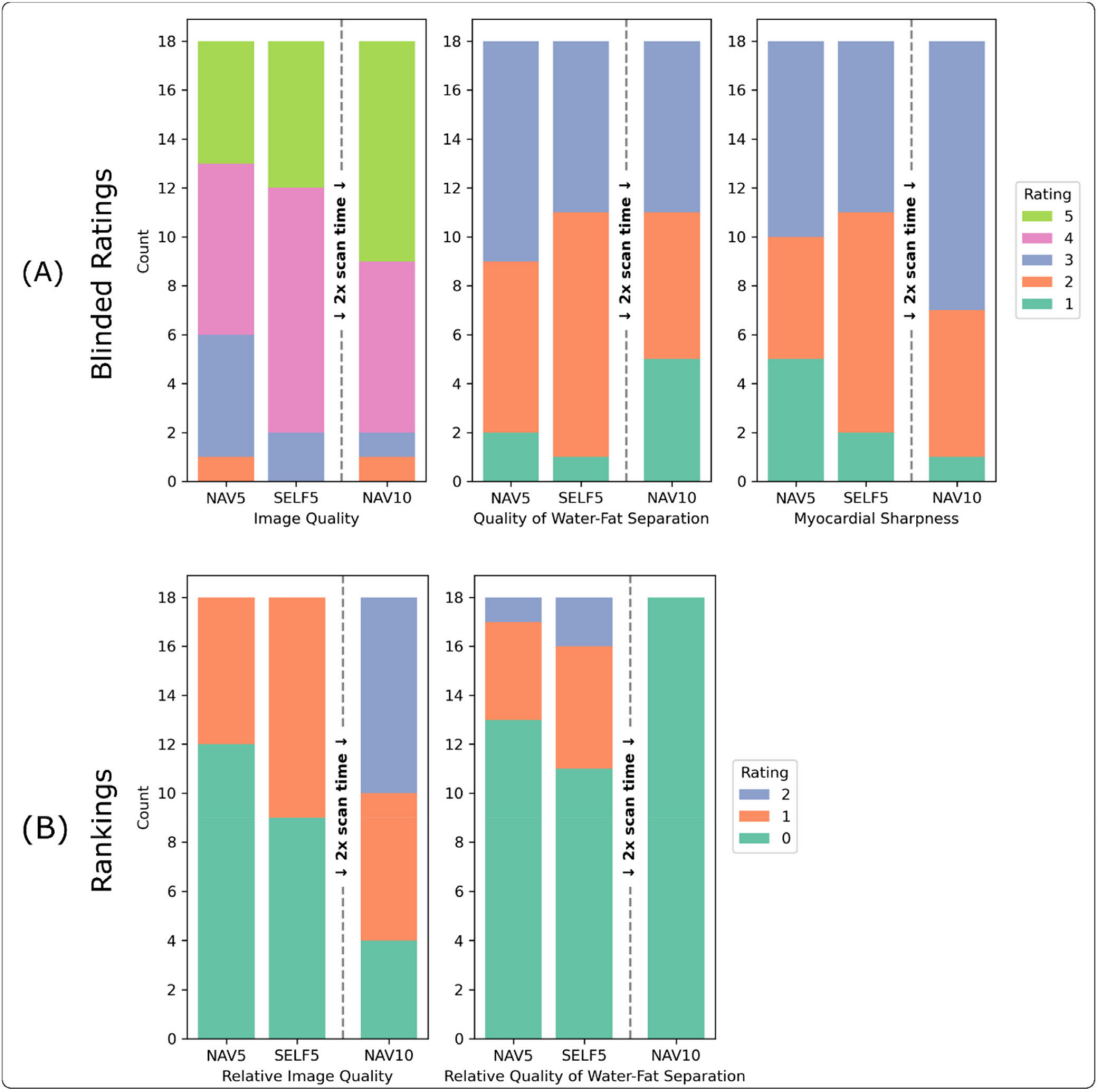
Results of the combined blinded expert rating (A). For image quality a trend can be observed where NAV10 performs best, followed by SELF5 and NAV5. Regarding water‐fat separation, NAV10 tended to coincide with larger water‐fat swaps. For myocardial sharpness there was only moderate inter‐rater agreement. Similar observations as for image quality can be made here. Result of the intra‐volunteer ranking with navigator obscurement (B). For relative image quality, a ranking of NAV5 < SELF5 < NAV10 can be inferred. However, for this statistic only poor inter‐rater reliability was observed. Regarding water‐fat separation, NAV10 performs worst. Higher rating scores correspond to more desirable image properties.

For the quality of the water‐fat separation, NAV10 had the biggest amount of large water‐fat swaps, but the Friedman test showed no statistically significant differences (*p* = 0.266). The inter‐rater correlation was 0.71 and mean scores were 2.39/2.33/2.11 for NAV5, SELF5 and NAV10 respectively. Regarding myocardial sharpness, a similar trend as for image quality could be observed. The inter‐rater correlation was 0.64 and the Friedman test showed no significant differences (*p* = 0.092). Mean scores were 2.17/2.28/2.56 for NAV5, SELF5, and NAV10 respectively. The summary of the combined expert rating is shown in Figure 7A. For image quality, a trend can be observed where NAV10 performed best, followed by SELF5 and NAV5, with respective mean scores of 3.89/4.22/4.33 for NAV5, SELF5 and NAV10 respectively and an inter‐rater correlation of 0.8. Only the NAV5 and NAV10 differences were statistically significant (*p* = 0.014).

The ranking results (Figure 7B) showed similar results for image quality as described above, however the inter‐rater correlation was 0.46. Significant differences between SELF5 and NAV10 (*p* = 0.025) and NAV5 and NAV10 (*p* = 0.006) were observed. Mean scores were 0.33/0.5/1.22 for NAV5, SELF5, and NAV10 respectively. For the quality of water‐fat separation, the inter‐rater correlation was 0.92. NAV10 was rated as the worst method. Differences between SELF5 and NAV10 were statistically significant (*p* = 0.042). Mean scores were 0.33/0.5/0.0 for NAV5, SELF5, and NAV10 respectively.

## 4 Discussion

In this work, a 3D Cartesian Dixon acquisition at 0.55 T with model‐based reconstruction, self‐gating and retrospective motion correction was evaluated in 18 volunteers and compared to a navigator‐gated reference. Despite the lower field strength, good image quality could be achieved.

While a bSSFP acquisition generally provides higher SNR, we selected a bipolar spoiled gradient echo readout in combination with Dixon for the following reasons. A bSSFP acquisition would require fat suppression, which for the large FoV used here is challenging to achieve homogeneously with spectral fat saturation techniques. Dixon enables fat separation, where the additional fat images can provide value, for instance in patients with myocardial fatty infiltrations. While improved B_0_ homogeneity at 0.55 T is beneficial for bSSFP, systems tend to exhibit limited gradient performance, resulting in longer echo times and potentially leading to banding artifacts. Although the combination of bSSFP with Dixon has been shown for 3D [27], it was achieved by configuring TR to an odd half‐multiple of the cycle time between fat and water and thus only acquiring a single echo per TR. While this is viable at 3 T, with a TR of 3.4 ms, at 0.55 T the same setup would require a TR of 6.2 or 18.6 ms at a 0.5 and 1.5 multiple respectively. Another problem inherent to bSSFP is its sensitivity to flow, off‐resonance and susceptibility artifacts [28].

In the respiratory‐resolved reconstruction (Figure 2), TV regularization was applied over the motion state dimension to address the limited k‐space coverage for each motion bin. This was the only stage in the reconstruction pipeline where this kind of regularization was incorporated. To ensure an accurate quantification of the motion, $\lambda_{\mathrm{TVT}}$ was carefully optimized. Four respiratory motion bins were selected for motion parameterization, as this configuration provided an acceptable balance between the accuracy of the estimated motion fields and the overall image quality. Although a higher number of native motion bins can improve reconstruction performance in high‐quality datasets, it was observed that, on average, such an increase led to degraded reconstruction outcomes. Therefore, we chose to instead retrospectively expand the number of motion states for each data set individually.

A key component of the proposed method was the model‐based water‐fat separation, whose corresponding signal model was described in Equation (3). Here, one of the primary challenges was the definition of the error phasors B, which were computed only once, using an image‐based Dixon algorithm (Figure 1C) and remained constant thereafter. Since regularization affects echo and water‐fat images differently, no regularization was applied to the respiratory‐resolved reconstruction, which served as this step's input. Following the computation of B, a smoothing operation was required to avoid local swaps. Further improvements might be achieved by updating B (and T) throughout the iterative optimization process [13]. The employed chemical shift model was empirically optimized for image‐based water‐fat reconstruction with a fixed second echo time ignoring a varying chemical shift throughout the short readout [13, 24].

Only certain components of the reconstruction framework were implemented for GPU computation (NVIDIA RTX A4000) leading to frequent data transfer between CPU and GPU. The computation time for the entire pipeline was therefore in the order of 50 min, consisting of 5 min for the respiratory‐resolved reconstruction, 20 min for the registration and computation of motion fields, 4 min for the motion‐corrected echoes, 1 min for the image‐based Dixon algorithm and 20 min for the model‐based reconstruction. While this might be acceptable in a research setting, further optimization is needed for clinical applicability.

Given the goal of achieving a 5‐min scan with a large FoV, substantial undersampling of k‐space was necessary. Compared to NAV5 or NAV10, SELF5 inherently required a distinct sampling distribution optimized for self‐gating and retrospective motion correction. The optimization of the sampling strategy required balancing two opposing objectives. First, a strong emphasis on central k‐space coverage for motion modeling was essential, which depended on the number of sampling patterns, the extent of the full sampled region, and the number of motion states. Ensuring sufficient central k‐space coverage in each motion bin was critical, as inadequate sampling in this region would result in poor motion parameter estimation. Second, adequate sampling of the k‐space periphery is important for the final reconstruction, which warranted the definition of a minimum sampling density. Insufficient peripheral sampling resulted in a blurred final reconstruction due to the lack of high‐frequency components.

Although the acquisition time of NAV10 was double that of SELF5 and NAV5, average scores for image quality in the expert rating differed only by 0.11 and 0.44 respectively. Although the total number of k‐space lines was similar between SELF5 and NAV10, their respective k‐space distribution differed fundamentally. NAV10 was expected to perform better, as its sampling pattern was exclusively optimized for the final reconstruction. Potential inaccuracies in the computation of the motion parameters may increase this difference in image quality. It should be noted that regarding image quality, inter‐rater agreement in the ranking was only moderate with a correlation of 0.46. This suggests only small differences between the methods regarding this criterion.

While the optimization of the sampling pattern for SELF5 was explored to some extent, several aspects remain to be further refined. These include fine‐tuning individual acceleration factors for each pattern, modifying the probability distribution, adjusting the degree of corner‐cropping, and optimizing the minimum sampling density.

To facilitate clinical applicability, predictable scan durations are essential. Variability in respiratory navigator efficiency results in greater standard deviation in scan duration in case of prospective gating, which could be observed in our experiments. Deviations of the total scan duration from the target scan time are not solely dependent on the respiratory acceptance rate. Additional contributing factors include missing or falsely identifying ECG triggers and heart rate variability during the scan. These factors explain why even SELF5 exhibited slight deviations from the target scan duration. The respiratory pattern of the volunteer is typically unknown and prone to large variations and thus poses an element of uncertainty. In the worst cases, NAV5 and NAV10 deviated from the estimated scan duration by +58.17% and +53.83%, respectively. Such deviations pose challenges for maintaining a tightly regulated clinical workflow and are expected to be even more consequential when imaging patients, as their respiratory acceptance rates are anticipated to be lower.

Prospective navigator gating can be the source of relative differences in image quality. Since for the navigator‐gated scans, the end‐expiratory state is assumed to remain constant throughout the acquisition, no retrospective motion correction is performed. In case of irregular breathing, a wider range of motion is accepted, yielding comparatively poorer results, as could be observed in Figure 5A. Image quality differences in navigator‐gated scans due to a changing end‐expiratory state are not just limited to inter‐subject cases but could also be observed in an intra‐subject context. This issue does not apply to SELF5, due to retrospective binning into motion states and subsequent motion correction. This also explains why, in the ranking (Figure 7), NAV10 did not always yield the best results.

Occasional water–fat swaps were observed in anatomical regions with expected blood flow, which is in line with previous work [29, 30]. In our analysis, NAV10 demonstrated a higher susceptibility to such swaps compared with NAV5 and SELF5. The underlying cause of this increased sensitivity warrants further investigation.

In Figure 5B, a case is shown where the image quality of SELF5 was rated less favorably compared to the reference method. The corresponding histograms of accepted respiratory positions are depicted, which showcase a regular and deep breathing pattern. While the low variability in the end‐expiratory position is optimal for the navigator‐gated approach, the wide range of motion constitutes a challenge for SELF5. The correlation of the respiratory motion range with the image quality rating was −0.22 for SELF5, suggesting a weak correlation. For large ranges of motion, more artificial motion states were introduced, which were derived via linear interpolation, likely leading to small errors.

Compared to the navigator‐gated reference, the self‐gated approach inherently avoids the navigator saturation artifact and thus offers a better visualization of vessels in the affected region (Figure 6). A notable example is the pulmonary veins, whose interaction with the navigator pulses has previously been shown to result in inflow artifacts [6]. Accurate depiction of the pulmonary veins is of clinical significance for various applications, such as the detection of anomalous pulmonary venous return [31], pre‐procedural planning of atrial fibrillation ablation [32], or post‐ablation assessment for pulmonary vein stenosis [33]. While we only showed the navigator saturation artifact in the pulmonary veins (Figure 6), its effects are visible along the saturation bands, including the liver.

Our study was verified across 18 different volunteers and is warranted to be further enhanced by increasing the sample size and including patients for a clinical evaluation. This would also lead to stronger statistics for the expert rating and scan time calculations.

## 5 Conclusion

This study presented a self‐gated 5‐min MRA acquisition incorporating model‐based water‐fat separation and motion correction. Evaluations at 0.55 T in 18 volunteers demonstrated on average better image quality than that of a prospectively navigator‐gated reference scan with a similar nominal duration. Compared to the reference method, the proposed method could be shown to offer several advantages, including a predictable scan time, 100% data exploitation, mitigation of navigator‐induced saturation effects, and greater robustness to irregular respiratory patterns.

## Funding

This work was partly funded by the German Research Foundation (GRK2260, BIOQIC). The project (22HLT02 A4IM) has received funding from the European Partnership on Metrology, co‐financed by the European Union's Horizon Europe Research and Innovation Programme and by the Participating States.

## Conflicts of Interest

Robert Stoll receives PhD funding from Siemens Healthineers AG. Michaela Schmidt is an employee of Siemens Healthineers AG. Daniel Giese is an employee of Siemens Healthineers AG. Marcel Dominik Nickel is an employee of Siemens Healthineers AG.

## Supporting information


**Video S1:** An animated GIF visualizing the motion fields for three volunteers, by overlaying the respiratory states with a regular grid morphed using the motion fields. For computational efficiency, the motion field was set to 0 outside a manually defined ROI of the torso.

**Video S2:** Video of coronal views across slices for all volunteers and methods showing fat and water images. Volunteer index added to the left; methods indicated atop each column.

**Table S1:** Unprocessed expert ratings for: image quality/quality of water fat separation/myocardial sharpness/relative image quality/relative quality of water fat separation. 0 means no rating was given.

## Data Availability

Research data are not shared.
