# Supplementary figures and images for: Respiratory Motion‐Corrected Model‐Based 3D Water‐Fat MRA of the Thorax at 0.55 T
Source: Magn Reson Med. 2026 Feb 4;95(6):3241–52. doi: 10.1002/mrm.70285 (PMC13049257; doi:10.1002/mrm.70285)

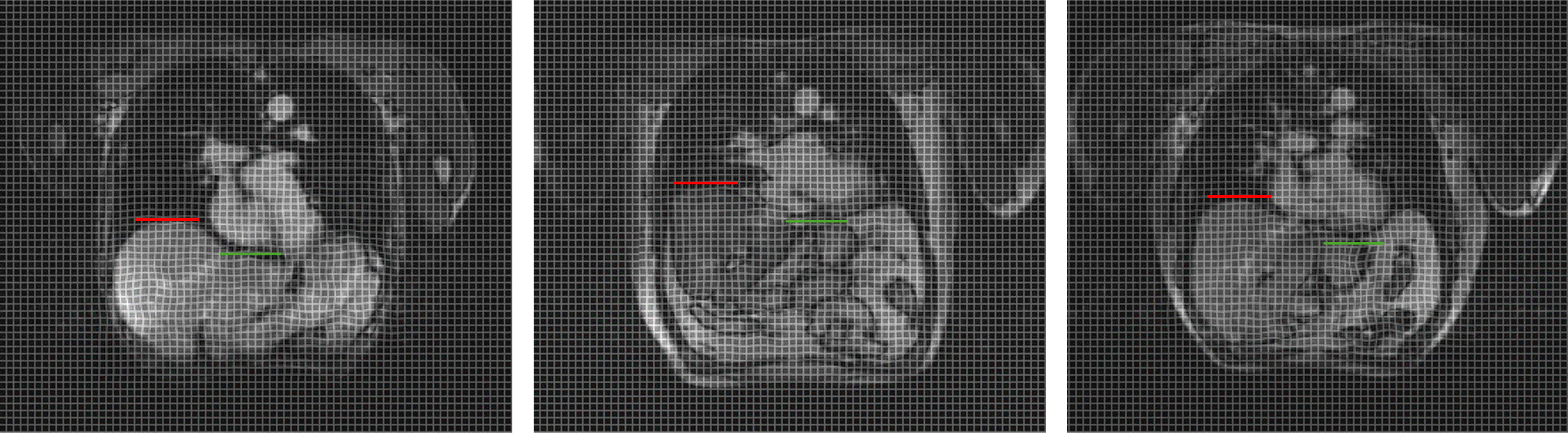

Supplement: Supplementary file 1 — Video S1: An animated GIF visualizing the motion fields for three volunteers, by overlaying the respiratory states with a regular grid morphed using the motion fields. For computational efficiency, the motion field was set to 0 outside a manually defined ROI of the torso. [file MRM-95-3241-s003.gif]
