# Supplementary material for: Respiratory Motion‐Corrected Model‐Based 3D Water‐Fat MRA of the Thorax at 0.55 T
Source: Magn Reson Med. 2026 Feb 4;95(6):3241–52. doi: 10.1002/mrm.70285 (PMC13049257; doi:10.1002/mrm.70285)
Supplement: Supplementary file 3 — Table S1: Unprocessed expert ratings for: image quality/quality of water fat separation/myocardial sharpness/relative image quality/relative quality of water fat separation. 0 means no rating was given. [file MRM-95-3241-s001.docx]

| Expert 2 | | | Expert 1 | | |  | Expert 2 | | | Expert 1 | | |  |
| --- | --- | --- | --- | --- | --- | --- | --- | --- | --- | --- | --- | --- | --- |
| SELF5 | NAV5 | NAV10 | SELF5 | NAV5 | NAV10 | Method | SELF5 | NAV5 | NAV10 | SELF5 | NAV5 | NAV10 | Method |
| 5/3/3/3/0 | 5/2/3/2/0 | 5/3/3/1/0 | 5/2/3/2/1 | 5/3/3/3/1 | 5/3/3/1/1 | Vol 10 | 5/3/3/1/0 | 4/3/2/2/0 | 4/3/2/3/0 | 5/3/3/1/1 | 4/2/2/2/1 | 4/2/2/2/1 | Vol 1 |
| 4/3/2/3/0 | 4/3/2/2/0 | 5/2/3/1/0 | 4/3/2/3/1 | 5/3/3/2/1 | 5/2/3/1/1 | Vol 11 | 5/3/3/1/0 | 4/3/2/3/0 | 5/3/3/2/0 | 5/3/1/2/1 | 3/3/1/3/1 | 4/3/3/1/1 | Vol 2 |
| 5/3/2/2/0 | 5/3/2/3/0 | 5/3/3/1/0 | 5/3/3/2/1 | 5/3/3/2/1 | 5/3/3/1/1 | Vol 12 | 5/3/3/3/0 | 4/3/2/2/0 | 5/3/3/1/0 | 5/2/3/1/1 | 5/2/3/2/1 | 5/3/3/1/1 | Vol 3 |
| 4/2/2/1/2 | 4/2/2/1/1 | 4/1/2/1/3 | 4/2/2/2/2 | 4/2/2/2/2 | 4/1/3/2/3 | Vol 13 | 4/3/2/3/0 | 3/3/2/2/0 | 4/3/2/1/0 | 4/3/2/3/2 | 3/2/2/2/2 | 4/3/2/1/2 | Vol 4 |
| 4/1/2/2/2 | 4/1/2/1/1 | 5/1/3/1/3 | 4/1/2/3/2 | 4/1/3/2/1 | 5/1/3/1/3 | Vol 14 | 4/3/2/3/0 | 4/2/2/1/0 | 4/3/2/2/0 | 4/2/2/1/1 | 4/2/3/3/2 | 4/2/2/2/2 | Vol 5 |
| 5/3/3/2/0 | 4/3/2/2/0 | 5/3/3/1/0 | 4/2/2/2/1 | 4/3/3/3/1 | 5/2/3/1/1 | Vol 15 | 5/3/3/3/0 | 4/3/3/2/0 | 5/3/3/1/0 | 4/2/2/2/1 | 4/3/2/2/1 | 5/3/3/1/1 | Vol 6 |
| 4/3/1/3/1 | 4/3/2/2/1 | 4/2/2/1/2 | 4/2/2/2/2 | 3/3/1/2/2 | 4/1/2/2/3 | Vol 16 | 4/3/2/3/0 | 4/3/2/1/0 | 3/2/1/2/0 | 3/3/1/1/1 | 3/3/1/2/1 | 3/2/2/3/1 | Vol 7 |
| 4/2/3/2/1 | 4/2/2/3/2 | 5/1/3/1/3 | 4/2/3/2/1 | 4/2/3/2/2 | 5/1/3/1/3 | Vol 17 | 5/3/3/2/0 | 5/3/2/3/0 | 5/3/3/1/0 | 5/3/3/2/1 | 5/3/2/3/1 | 5/3/3/1/1 | Vol 8 |
| 4/3/2/1/1 | 1/3/1/2/1 | 1/2/1/2/2 | 4/2/2/2/1 | 3/2/1/2/1 | 2/2/1/2/2 | Vol 18 | 3/3/1/3/1 | 3/1/1/2/3 | 3/1/2/1/2 | 3/2/1/3/1 | 3/1/1/2/3 | 4/1/2/1/3 | Vol 9 |

Table S1: Unprocessed expert ratings for: image quality/quality of water fat separation/myocardial sharpness/relative image quality/relative quality of water fat separation. A rating of 0 means no rating was given. For the first three metrics, higher rating scores correspond to more desirable image properties; for the last two metrics lower ranking scores correspond to more desirable image properties.

| Expert 2 | | | Expert 1 | | |  | Expert 2 | | | Expert 1 | | |  |
| --- | --- | --- | --- | --- | --- | --- | --- | --- | --- | --- | --- | --- | --- |
| SELF5 | NAV5 | NAV10 | SELF5 | NAV5 | NAV10 | Method | SELF5 | NAV5 | NAV10 | SELF5 | NAV5 | NAV10 | Method |
| 5/3/3/3/0 | 5/2/3/2/0 | 5/3/3/1/0 | 5/2/3/2/1 | 5/3/3/3/1 | 5/3/3/1/1 | Vol 10 | 5/3/3/1/0 | 4/3/2/2/0 | 4/3/2/3/0 | 5/3/3/1/1 | 4/2/2/2/1 | 4/2/2/2/1 | Vol 1 |
| 4/3/2/3/0 | 4/3/2/2/0 | 5/2/3/1/0 | 4/3/2/3/1 | 5/3/3/2/1 | 5/2/3/1/1 | Vol 11 | 5/3/3/1/0 | 4/3/2/3/0 | 5/3/3/2/0 | 5/3/1/2/1 | 3/3/1/3/1 | 4/3/3/1/1 | Vol 2 |
| 5/3/2/2/0 | 5/3/2/3/0 | 5/3/3/1/0 | 5/3/3/2/1 | 5/3/3/2/1 | 5/3/3/1/1 | Vol 12 | 5/3/3/3/0 | 4/3/2/2/0 | 5/3/3/1/0 | 5/2/3/1/1 | 5/2/3/2/1 | 5/3/3/1/1 | Vol 3 |
| 4/2/2/1/2 | 4/2/2/1/1 | 4/1/2/1/3 | 4/2/2/2/2 | 4/2/2/2/2 | 4/1/3/2/3 | Vol 13 | 4/3/2/3/0 | 3/3/2/2/0 | 4/3/2/1/0 | 4/3/2/3/2 | 3/2/2/2/2 | 4/3/2/1/2 | Vol 4 |
| 4/1/2/2/2 | 4/1/2/1/1 | 5/1/3/1/3 | 4/1/2/3/2 | 4/1/3/2/1 | 5/1/3/1/3 | Vol 14 | 4/3/2/3/0 | 4/2/2/1/0 | 4/3/2/2/0 | 4/2/2/1/1 | 4/2/3/3/2 | 4/2/2/2/2 | Vol 5 |
| 5/3/3/2/0 | 4/3/2/2/0 | 5/3/3/1/0 | 4/2/2/2/1 | 4/3/3/3/1 | 5/2/3/1/1 | Vol 15 | 5/3/3/3/0 | 4/3/3/2/0 | 5/3/3/1/0 | 4/2/2/2/1 | 4/3/2/2/1 | 5/3/3/1/1 | Vol 6 |
| 4/3/1/3/1 | 4/3/2/2/1 | 4/2/2/1/2 | 4/2/2/2/2 | 3/3/1/2/2 | 4/1/2/2/3 | Vol 16 | 4/3/2/3/0 | 4/3/2/1/0 | 3/2/1/2/0 | 3/3/1/1/1 | 3/3/1/2/1 | 3/2/2/3/1 | Vol 7 |
| 4/2/3/2/1 | 4/2/2/3/2 | 5/1/3/1/3 | 4/2/3/2/1 | 4/2/3/2/2 | 5/1/3/1/3 | Vol 17 | 5/3/3/2/0 | 5/3/2/3/0 | 5/3/3/1/0 | 5/3/3/2/1 | 5/3/2/3/1 | 5/3/3/1/1 | Vol 8 |
| 4/3/2/1/1 | 1/3/1/2/1 | 1/2/1/2/2 | 4/2/2/2/1 | 3/2/1/2/1 | 2/2/1/2/2 | Vol 18 | 3/3/1/3/1 | 3/1/1/2/3 | 3/1/2/1/2 | 3/2/1/3/1 | 3/1/1/2/3 | 4/1/2/1/3 | Vol 9 |

Table S1: Unprocessed expert ratings for: image quality/quality of water fat separation/myocardial sharpness/relative image quality/relative quality of water fat separation. A rating of 0 means no rating was given.
